# Sino-Austrian High-Tech Acupuncture Network—Annual Report 2015

**DOI:** 10.3390/medicines4010013

**Published:** 2017-02-24

**Authors:** Gerhard Litscher

**Affiliations:** Research Unit for Complementary and Integrative Laser Medicine, Research Unit of Biomedical Engineering in Anesthesia and Intensive Care Medicine, and TCM Research Center Graz, Medical University of Graz, Auenbruggerplatz 29, 8036 Graz, Austria; gerhard.litscher@medunigraz.at; Tel.: +43-316-385-13907; Fax: +43-316-385-13908

**Keywords:** high-tech acupuncture, network, annual report, China, Austria

## Abstract

The Sino-Austrian High-Tech Acupuncture Research Network was founded in 2005 and has been growing ever since. The network comprises many partners from China and is highly involved in research and education activities. This report introduces the network’s activities in the year 2015.

Within 2015, the Sino-Austrian High-Tech Acupuncture Network has grown very quickly. The network was founded in 2005 by Prof. DDr. Gerhard Litscher from Medical University of Graz and comprises many partners from China. In the following, some milestones and publications [1,2,3,4,5,6,7,8,9,10,11,12,13,14,15,16,17,18,19,20,21,22,23,24,25,26,27,28,29,30,31,32,33,34] from the year 2015 are listed chronologically:

**9 January 2015:** Meeting and discussion with the Dean of Beijing University of Chinese Medicine (School of Acupuncture and Moxibustion) Prof. Dr. Zhao Baixiao about further cooperation on moxibustion projects (Figure 1).

**9 January 2015:** Scientific Cooperation with the Military Acupuncture Training Center at Chinese People’s Liberation Army General Hospital in Beijing (Figure 2).

**9 January 2015:** Meeting of Sino-Austrian auricular acupuncture project leaders in Beijing, China, 9 and 15 January 2015 (Figure 3). Topic: future project discussion.

**12 January 2015:** Lecture at Hubei University of Chinese Medicine Wuhan, China (Figure 4).

**12 January 2015:** Hubei University of Chinese Medicine VIP reception room, Wuhan, China (Figure 5).

**13 January 2015:** Editor’s meet in Wuhan, *Medicines* Wuhan, China (Figure 6).

**14 and 15 January 2015:** Eurasia-Pacific Uninet (EPU), Beijing, China (Figure 7).

**15 January 2015:** Peking University Health Science Center, Department Integration of Chinese and Western Medicine, Beijing, China. Project discussion about microcirculation research (Figure 8).

**16 January 2015:** Project meeting: Beijing University of Science and Technology Beijing, China. Joint transcontinental basic research, biosignal analysis (Figure 9).

**16 January 2015:** Editor’s meet in Beijing, *Medicines* Beijing, China (Figure 10).

**25 and 26 February 2015:** Meeting, German-Chinese Research Foundation for TCM. DCFG (Deutsch-Chinesische Forschungsgemeinschaft)-TCM, Heidelberg Institute for Chinese Medicine (Figure 11).

**18 May 2015:** Beijing University of Chinese Medicine, School of Acupuncture-Moxibustion and Tuina (Figure 12).

**18 May 2015:** Editor’s Meet in Beijing, *Medicines* Beijing, China, 18 May 2015. Gerhard Litscher: Editor-in-chief of *Medicines* (Figure 13).

**20 May 2015:** Hubei Provincial Collaborative Innovation Center of Preventive Treatment by Acupuncture and Moxibustion (Figure 14).

**22 and 24 May 2015:** 2^nd^ Annual World Congress, 2015 High-Tech Acupuncture & Integrative Medicine and 9^th^ International Workshop of the German-Chinese Research Foundation for Traditional Chinese Medicine (DCFG (Deutsch-Chinesische Forschungsgemeinschaft)-TCM), Hangzhou, China, 22–24 May 2015 (Figure 15 and Figure 16).

Lecturers from many different countries and regions from all over the world, like Armenia, Australia, Austria, Belgium, Brazil, Canada, China, Croatia, Denmark, Ecuador, Estonia, France, Germany, Greece, Hong Kong, Iceland, India, Italy, Israel, Japan, Korea, New Zealand, Oman, Poland, Russia, Saudi Arabia, Singapore, Sweden, Spain, Switzerland, South Korea, Taiwan, Thailand, the United Kingdom and the United States, have presented their research results. Altogether, 170 speakers have presented their lectures, and during the three days, we had 310 participants. Fifty poster presentations completed the scientific program.

**25 May 2015:** Baoshan Hospital affiliated to Shanghai University of Traditional Chinese Medicine Shanghai, China (Figure 17).

**25 May 2015:** Shuguang Hospital affiliated to Shanghai University of Traditional Chinese Medicine Shanghai, China, 26 May 2015. 1st Sino-Austrian TCM-Meeting, Shanghai. Gerhard Litscher, Daniela Litscher, Ingrid Gaischek and representatives of Shuguang Hospital affiliated with Shanghai University of TCM (Figure 18).

**1 July 2015:** Meeting in Vienna with Vice-President of BIT (BIT Congress Inc.) Dr. Haobo Mei (Figure 19).

BIT Congress, Inc., a BIT Group Global company, is the largest professional conference organizing and business operating company in China. BIT has over 500 employees, 60% among which have masters and Ph.D. degrees. Founded in 2003, BIT has organized more than 300 conferences at home and abroad and has expanded its operations to include such areas as biotechnology, pharmaceutical, environmental protection, new energy, advanced materials, IT, marine technology, economy, etc. BIT Congress Inc. has invited over 45 Nobel Prize Laureates and more than 100,000 experts and entrepreneurs to participate in the programs.

**5 and 6 September 2015:** International Conference on Health, Healthcare and Eco-civilisation, London, UK (Figure 20).

**15 October 2015:** Editor’s meet in Beijing, *Medicines*, Beijing, China (Figure 21).

**16 October 2015:** Interview for China Net of TCM, ‘Acupuncture in Austria’. Beijing, China (Figure 22).

**16 and 17 October 2015:** 10 Years successful cooperation with China Academy of Chinese Medical Sciences (CACMS). Final meeting of Sino-Austrian TCM joint research project (second phase) prevention and early intervention of chronic diseases with TCM, Beijing, China (Figure 23).

**19 October 2015:** Board meeting at Xiyuan Hospital. *Chinese Journal of Integrative Medicine* (*CJIM*), Beijing, China. Gerhard. Litscher: Member of the Editorial Board of CJIM (Figure 24).

**20 October 2015:** Project meeting: Beijing University of Science and Technology, Beijing, China (Figure 25).

**21 October 2015:** Post-meeting of Sino-Austrian acupuncture project leaders: Prevention and Early Intervention of Chronic Diseases with TCM (Figure 26).

**16 and 21 October 2015:** Tu Youyou, Nobel Laureate in Medicine 2015 and relationships with the research at the TCM Research Center at the Medical University of Graz, Beijing, China.

For the first time in history, the Nobel Prize in Medicine 2015 is awarded to a researcher who is specialized in Traditional Chinese Medicine. The Chinese Tu Youyou received this highest award in medicine for her excellent work in malaria research. Tu Youyou and her team found out that artemisinin, the active ingredient of the medicinal herb “sweet wormwood”, represents an effective malaria therapy. Photos of the research on moxibustion using *Artemisia* are also presented here; these studies were carried out at the TCM Research Center Graz, partly in close cooperation with the “China Academy of Chinese Medical Sciences”, the institution of the Nobel Prize winner (Figure 27).

**7 November 2015:** International Academic Development Congress for the 60th Anniversary of China Academy of Chinese Medical Sciences. Lecture Hall of Conference Building, Beijing Conference Center, 7 November 2015. Nine hundred participants. Representatives for Europe from Austria: Vicerector Prof. Irmgard Lippe and Prof. Gerhard Litscher (Figure 28).

**18 November 2015:** World Congress of Regenerative Medicine & Stem Cell, 2015, Shanghai, China, 17–19 November 2015 (Figure 29).

**19 November 2015:** Shanghai University of TCM (Figure 30).

**20 November 2015:** Beijing Dance Academy. Eurasia Pacific Uninet, Beijing, China (Figure 31).

**20 November 2015:** Peking University, Health Science Center. Eurasia Pacific Uninet (EPU), PhD interviews (Figure 32).

**21 and 22 November 2015:** PhD Workshop China 2015. Swissotel, Eurasia Pacific Uninet, Beijing, China (Figure 33).

**18 December 2015:** MDPI academic editors 2015 annual banquet in Beijing, China (Figure 34).

**20 December 2015:** People’s Liberation Army General Hospital, Beijing, China (Figure 35).

**22 December 2015:** World Health Organization, CACMS (China Academy of Chinese Medical Sciences) (Figure 36).

**22 December 2015:** 60th Anniversary of China Academy of Chinese Medical Sciences. Nobel Laureate in Medicine 2015, Prof. Tu Youyou, Beijing, China (Figure 37).

**22 December 2015:** 60th Anniversary of China Academy of Chinese Medical Sciences (Figure 38).

## Figures and Tables

**Figure 1:**
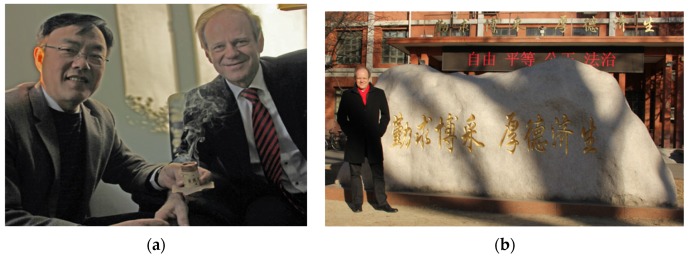
Dean Prof. Dr. Zhao Baixiao (**a** left) and Prof. Gerhard Litscher (**a** right) at Beijing University of Chinese Medicine (**b**), Beijing, China, 9 January 2015.

**Figure 2:**
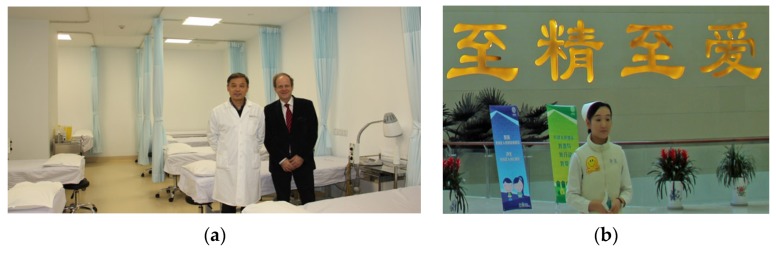
Prof. Shi Xian, Chairman of the Military Acupuncture Training Center (**a** left) and Prof. Gerhard Litscher (**a** right), Beijing, China (301 Hospital (**b**)), 9 January 2015.

**Figure 3:**
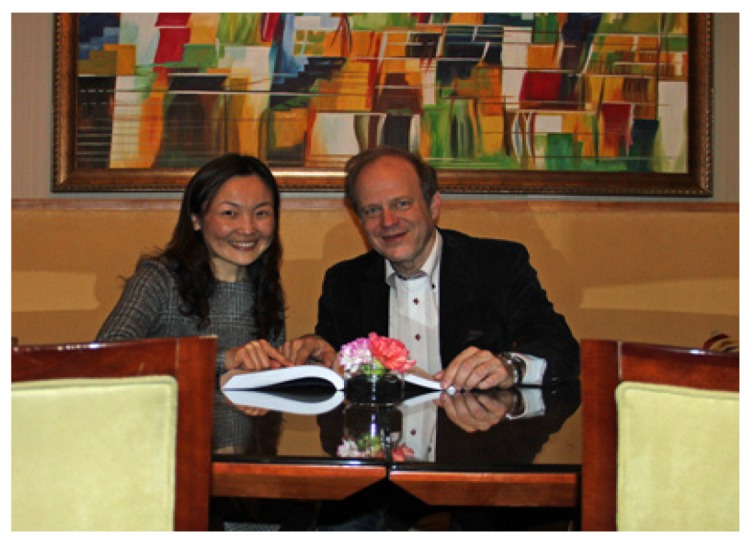
Prof. Rong Peijing, Vice Director of Auricular Acupuncture Professional Committee, China Association of Acupuncture and Moxibustion (CAAM), Professor at Institute of Acupuncture and Moxibustion at China Academy of Chinese Medical Sciences (left) and Prof. Gerhard Litscher (right). Beijing, China, 9 January 2015.

**Figure 4:**
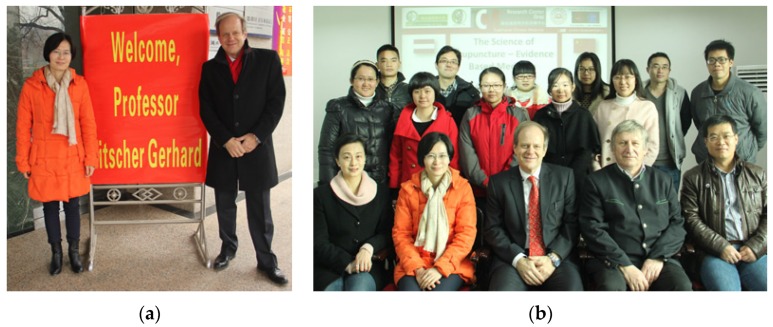
Prof. Liang Fengxia, Director of the Institute of Acupuncture and Moxibustion, Hubei University of Chinese Medicine (**a** left), Prof. Wolf-Dieter Rausch, President of Eurasia Pacific Uninet (**b** first row, second from right) and Prof. Gerhard Litscher (**b** first row, middle), Wuhan, 12 January 2015.

**Figure 5:**
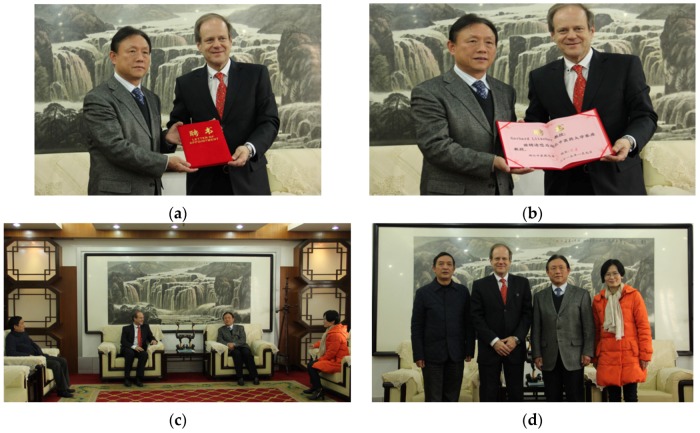
Prof. Wang Hua, President of Hubei University of Chinese Medicine (**a**,**b** left) with Prof. Gerhard Litscher (**a**,**b** right), Wuhan, China, 12 January 2015. Gerhard Litscher, Visiting Professor at Hubei University of Chinese Medicine (**a**–**d**).

**Figure 6:**
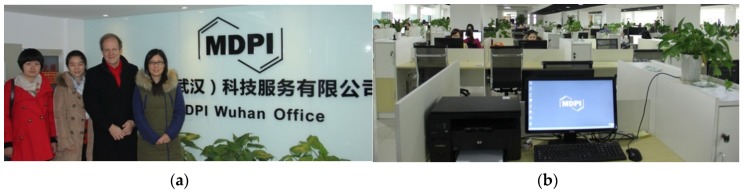
Gerhard Litscher: Editor-in-chief of *Medicines* (**a** middle right) (MDPI Wuhan Office (**b**)).

**Figure 7:**
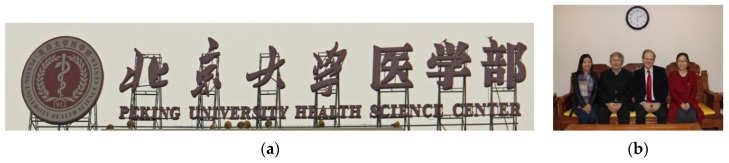
Interviews performed by: Prof. Wolf-Dieter Rausch, President of Eurasia-Pacific Uninet (EPU) (**b** middle left) and Prof. Gerhard Litscher (**b** middle right), at Peking University (**a**), 14 and 15 January 2015.

**Figure 8:**
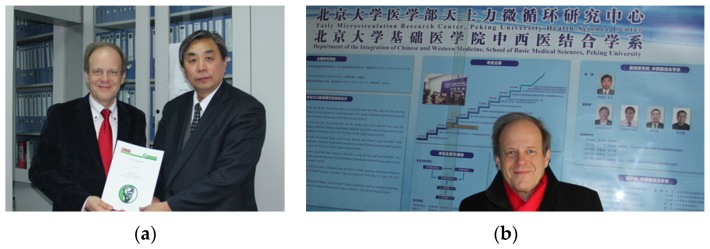
Prof. Han Jing-Yan, Dean and Chairman, Department Integration of Chinese and Western Medicine, Tasly Microcirculation Research Center (**a** right) and Prof. Gerhard Litscher (**a** left) at Peking University Health Science Center (**b**), 15 January 2015.

**Figure 9:**
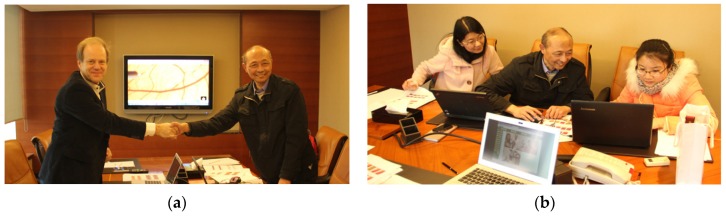
Prof. Min Lequan (**a** right, **b** middle)) and Prof. Gerhard Litscher (**a** left), Beijing, China, 16 January 2015.

**Figure 10:**
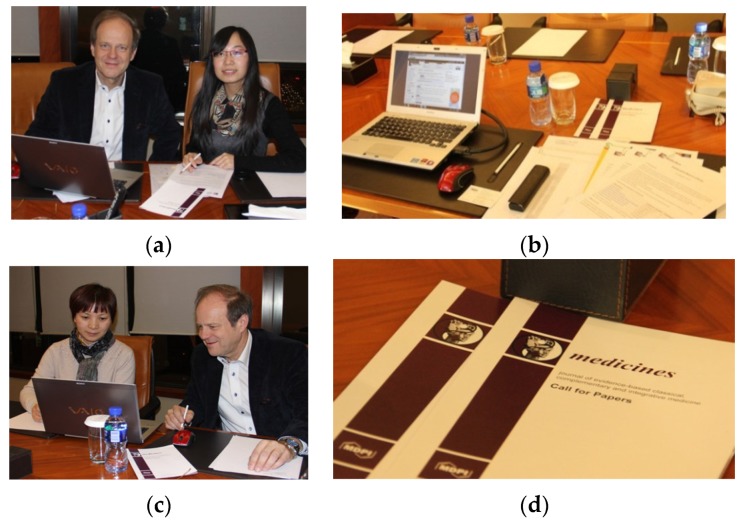
Editor-in-chief of *Medicines* (**b**,**d**) Prof. Gerhard Litscher (**a** left, **c** right) with assistant editor Ms. Cheng Xiaoyan (**a** right) and associate editor Prof. Gao Xinyan (**c** left), Beijing, China, 16 January 2015.

**Figure 11:**
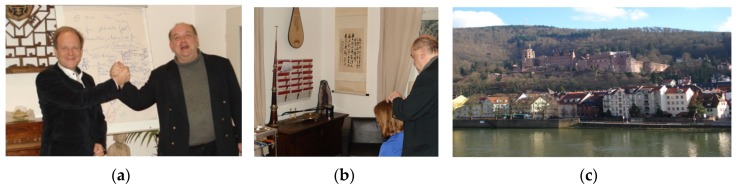
DCFG (Deutsch-Chinesische Forschungsgemeinschaft)-TCM President Prof. Dr. Johannes Greten, University Porto (**a**, right, **b** left)) and DCFG-TCM German Vice President Prof. Gerhard Litscher (**a** left), Heidelberg (**c**), Germany, 25 and 26 February 2015.

**Figure 12:**
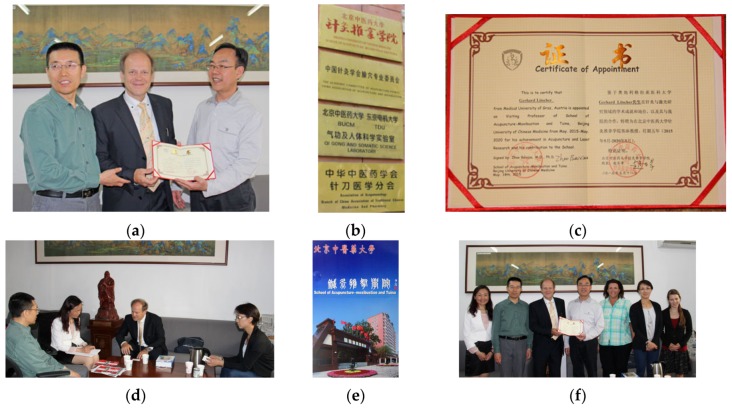
Prof. Zhao Baixiao, Dean of School of Acupuncture-Moxibustion and Tuina (**e**) of Beijing University of Chinese Medicine (**a** right, **f** middle), Prof. Zhou Liqun, Secretary General, Auricular Branch of China Association of Acupuncture (**a**,**d** left), Prof. Rong Peijing (**d**, second from left, **d** left) with Prof. Gerhard Litscher (**a**,**d** middle, **f** middle left)and his team, Beijing, China, 18 May 2015. Gerhard Litscher, Visiting Professor at Beijing University of Chinese Medicine (**a**–**f**).

**Figure 13:**
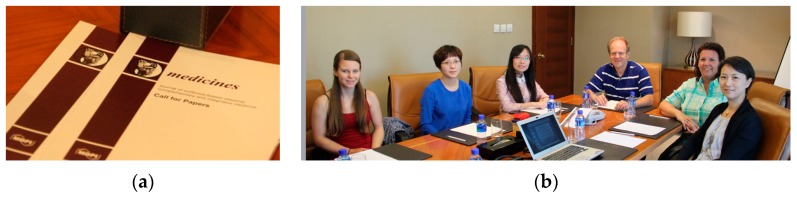
Editor-in-chief of *Medicines* (**a**) Prof. Gerhard Litscher (**b** middle right)) with assistant editor Ms. Cheng Xiaoyan (**b** middle left)and associate editor Prof. Gao Xinyan (**b** second from left), Daniela Litscher MSc (**b** left), Ingrid Gaischek MSc (**b** second from right) and Prof. Wang Lu (**b** right), Beijing, China.

**Figure 14:**
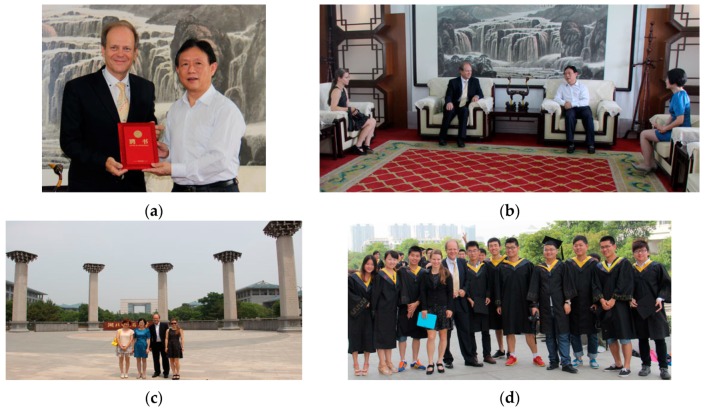
Prof. Wang Hua, President of Hubei University of Chinese Medicine (**a** right, b middle right), Gerhard Litscher, Honorary Professor (**a** left, **b** middle left), Prof. Liang Fengxia (**b** right), Dr. Daniela Litscher (**b** left) at Hubei University of Chinese Medicine (**c**,**d**), Wuhan, China, 20 May 2015.

**Figure 15:**
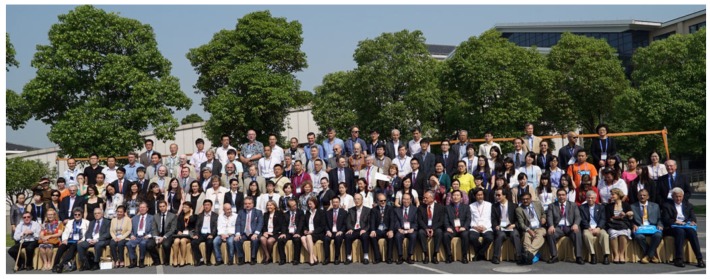
Venue: Hangzhou Blossom Water Museum Hotel, China. Congress Chair: Gerhard Litscher, Medical University of Graz, Austria, Europe. Congress Co-chair: Lu Wang, Medical University of Graz, Austria, Europe. Executive Chair: Xiaodan Mei, BIT (BIT Congress Inc.), Dalian, China.

**Figure 16:**
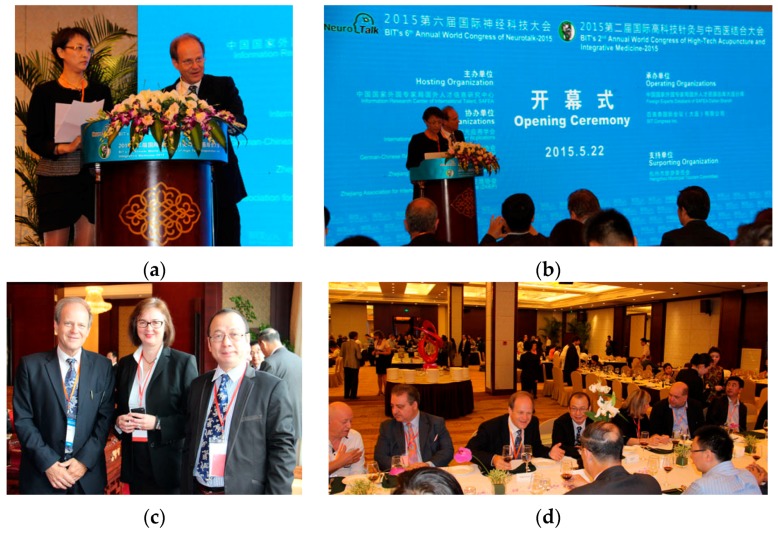
Opening Ceremony: Congress Chair Gerhard Litscher (**a**,**b** right, **c** left, **d** middle), Austrian Consul General from Shanghai Silvia Neureiter (**c** middle), Executive Chair President Xiaodan Mei (**c** right), and Congress Co-Chair Lu Wang (**a**,**b** left).

**Figure 17:**
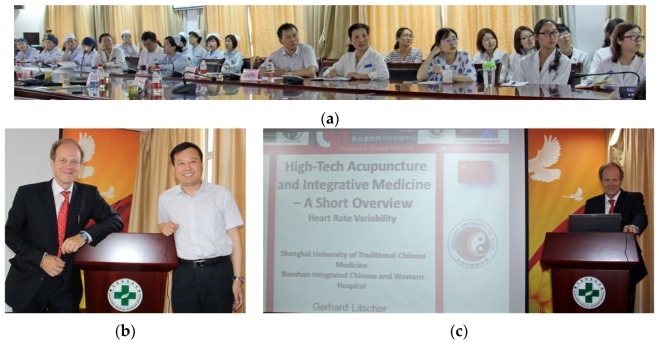

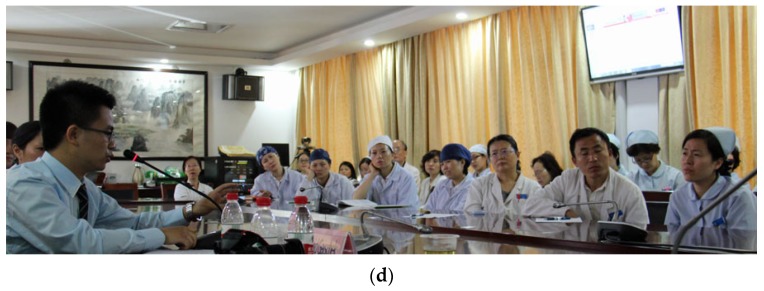
Prof. Ding Ren, Director and Surgeon of the Baoshan Hospital, Shanghai (**b** right), Prof. Gerhard Litscher (**b** left, **c**) and Dr. Liu Zhidan (**d** left) at Baoshan Hospital affiliated to Shanghai University of Traditional Chinese Medicine (**a**–**d**) 25 May 2015.

**Figure 18:**
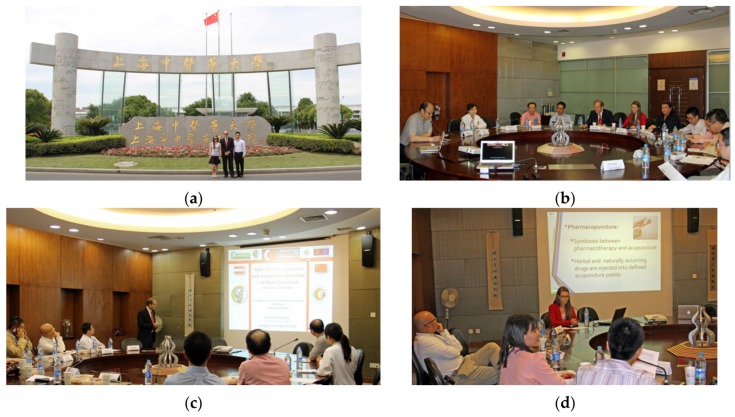
Prof. Shen Weidong, Director of Acupuncture Department (**c** left), Prof. Pan Weidong, Editor-in-chief of Integrative Medicine International (**b** left), Dr. Liu Zhidan (**a** left), Prof. Zhimin Fei, chairman and head of Neurosurgical Department and the team of the TCM Research Center at Medical University of Graz (**a**–**d**) in Shanghai.

**Figure 19:**
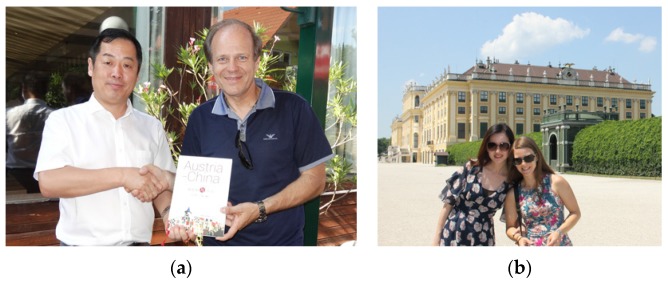
Dr. Haobo Mei, Vice President of BIT Congress Inc. (Dalian, China) (**a** left) and Prof. Gerhard Litscher (**a** right) in Vienna (Schönbrunn Palace (**b**)).

**Figure 20:**
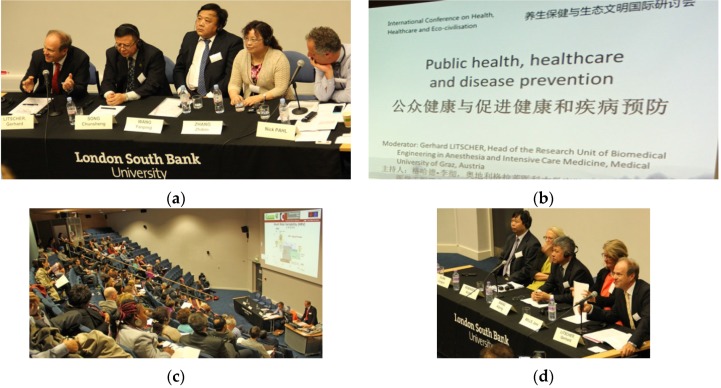
Invited lecture from Prof. Gerhard Litscher (Graz, Austria) (**a** left, **d** right) on 'The Sino-European high-tech acupuncture network. A contribution to the modernization of TCM in view of the demographic changes of the 21st century’ and Moderator of Session 3 in London, UK (**b**,**c**).

**Figure 21:**
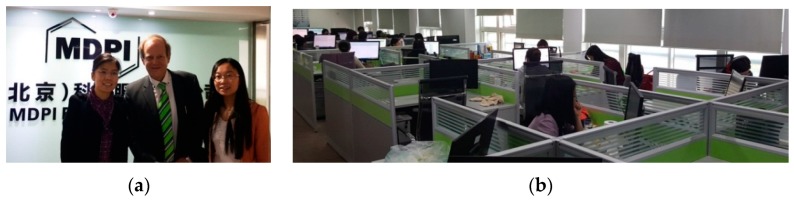
Editor-in-chief Prof. Gerhard Litscher (**a** middle) with the editorial team of *Medicines* in the office in Beijing, China (**b**).

**Figure 22:**
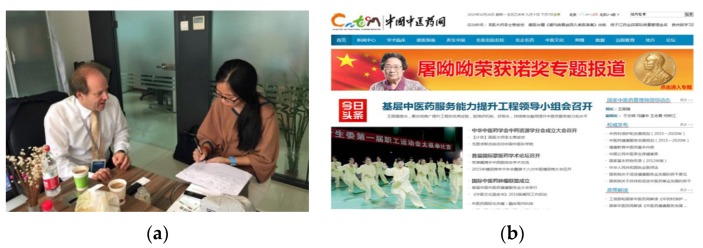
Zhao Weiting, China Net of Traditional Chinese Medicine (**a** right) and Prof. Gerhard Litscher (**a** left) during the 1-h interview for China Net of TCM (**b**) in Beijing, China, 16 October 2015.

**Figure 23:**
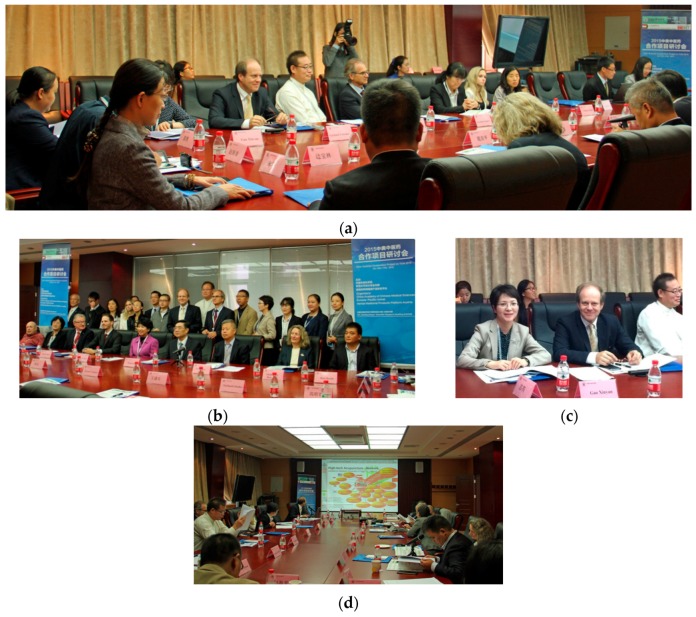
Presentation of the Sino-Austrian acupuncture research network results (**a**–**d**). Beijing, China, 16 October 2015.

**Figure 24:**
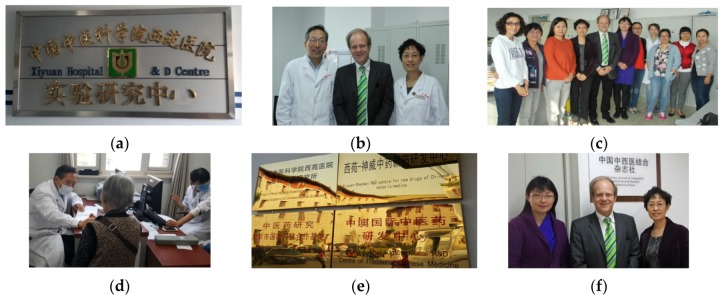
Prof. Shi Dazhuo, Vice President of Xiyuan Hospital (**a**,**e**) affiliated to China Academy of Chinese Medical Sciences (**b**,**d** left), Dr. Wang Weixia (**b**,**f** right), Dr. Guo Yan (**f** left), both senior editors of Chinese Journal of Integrative Medicine and Prof. Gerhard Litscher (**b**,**c**,**f** middle), Member of the Editorial Board.

**Figure 25:**
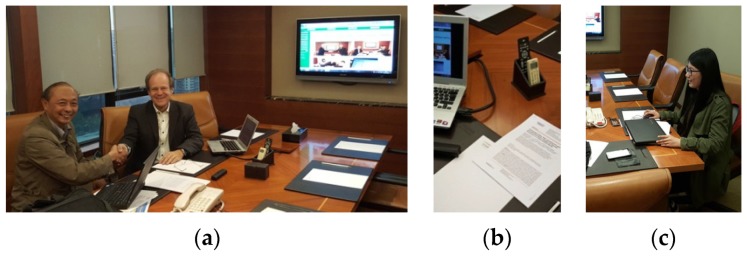
Joint transcontinental basic research, biosignal analysis (**b**). Prof. Min Lequan (**a** left), Dr. Li Min (**c**) and Prof. Gerhard Litscher (**a** right), Beijing, China, project discussion.

**Figure 26:**
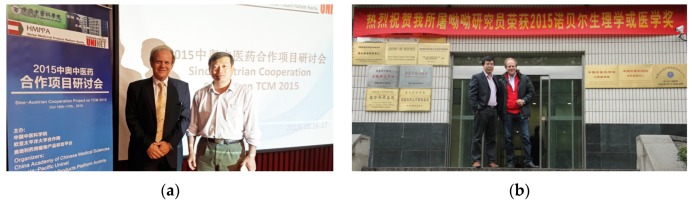
Prof. Zhang Weibo, Head of Department of Biomedical Engineering, Institute of Acupuncture and Moxibustion, China Academy of Chinese Medical Sciences (**a** right), Assoc. Prof. Dr. Wang Guangjun (**b** left) and Prof. Gerhard Litscher (**a** left, **b** right), Beijing, China, 21 October 2015.

**Figure 27:**
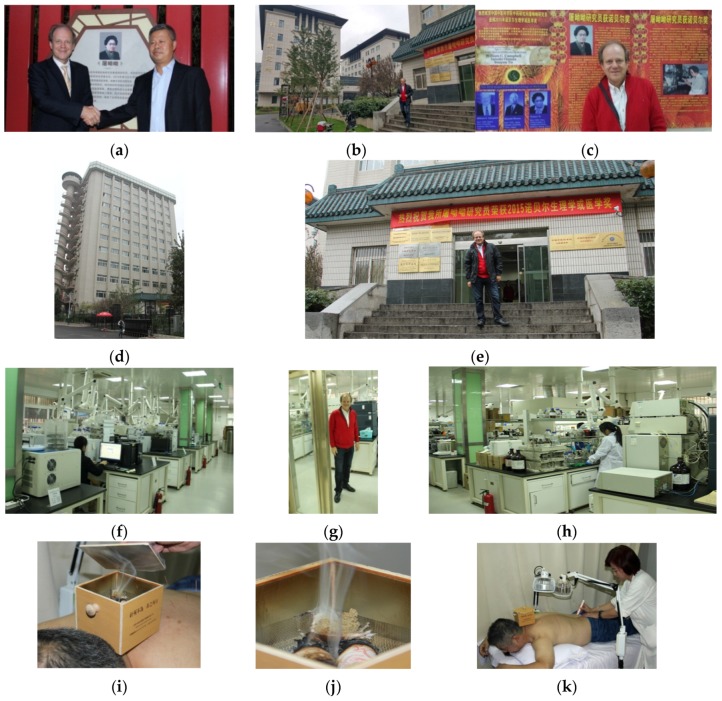
Vice President of China Academy of Chinese Medical Sciences (CACMS) Professor Fan Jiping (**a** right) and Prof. Gerhard Litscher (**a** left, **c**,**e**,**g**) in Beijing (16 October 2015). Laboratory and office of the Nobel Prize Winner Tu Youyou at CACMS in Beijing, 21 October 2015 (**b**–**h**). Joint moxibustion research using *Artemisia* between the TCM Research Center at the Medical University of Graz and the China Academy of Chinese Medical Sciences, the institution of the Nobel prize winner 2015 Tu Youyou (**i**–**k**). Beijing, China, 21 October 2015.

**Figure 28:**
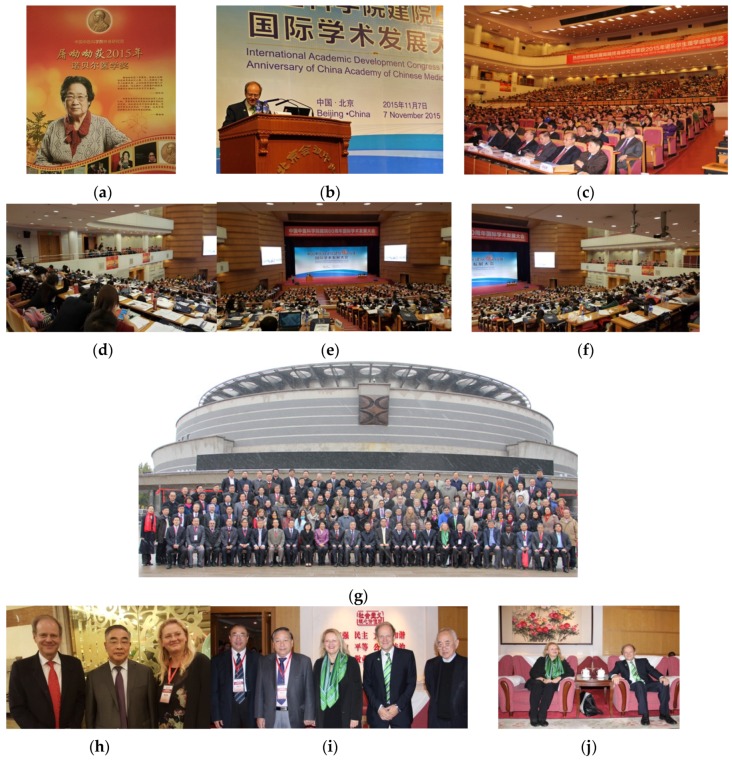
60th Anniversary of China Academy of Chinese Medical Sciences (**a**–**j**). Prof. Zhang Boli, President of China Academy of Chinese Medical Sciences (**h** middle), Vice Rector Prof. Irmgard Lippe (**h** right, **j** left) and Prof. Gerhard Litscher (**b,h** left, **j** right), both Medical University of Graz, Austria, Beijing, China, 7 November 2015.

**Figure 29:**
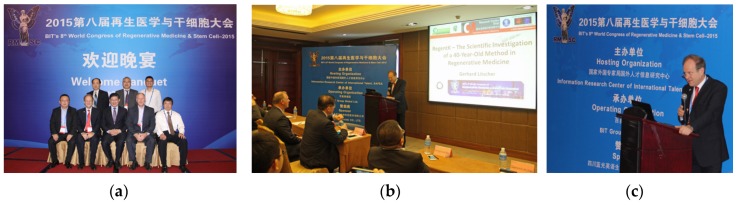

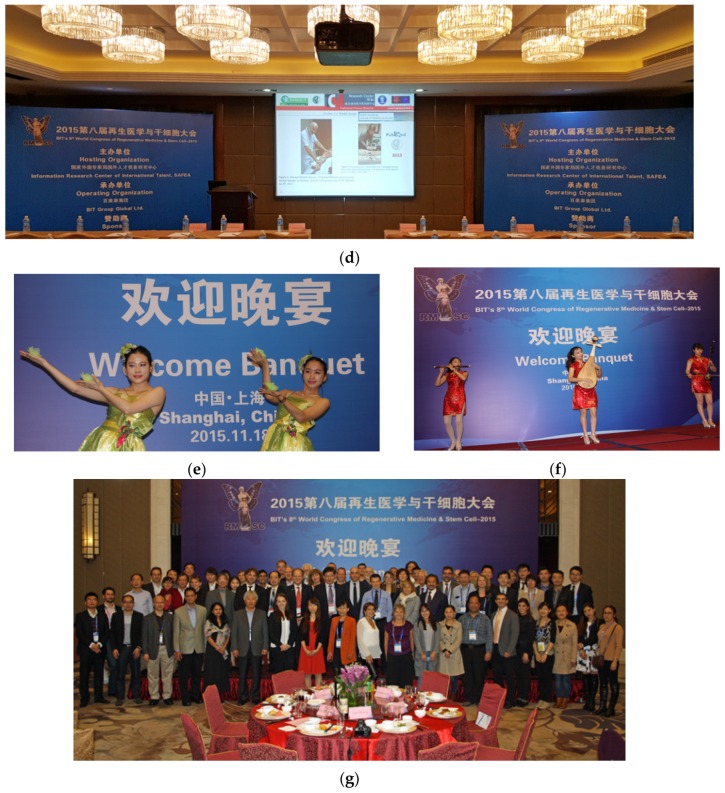
World Congress of Regenerative Medicine & Stem Cell, 2015 (**a**–**g**). Keynote Lecture, ‘REGENTK’ (Regeneration Therapy by Khalifa) from Prof. Gerhard Litscher (**b**,**c**), Medical University of Graz on 18 November 2015 in Shanghai, China.

**Figure 30:**
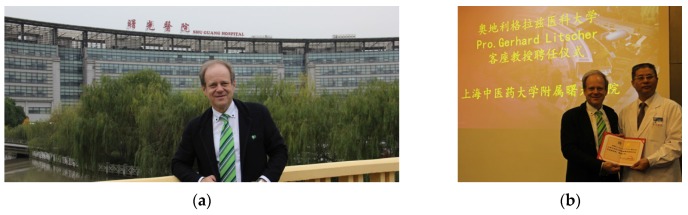

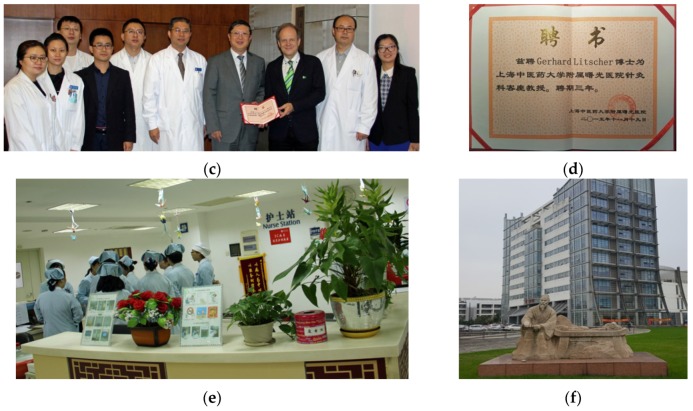
Gerhard Litscher (**a**,**b** left), Visiting Professor (**c**,**d**) at Shanghai University of TCM, Shu Guang Hospital, Institute of Acupuncture, Shanghai (**e**,**f**), China, 19 November 2015.

**Figure 31:**
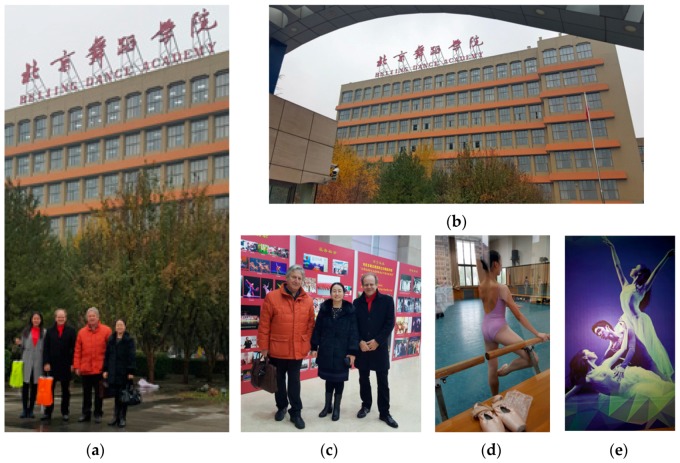
Beijing Dance Academy (**a**–**e**). Eurasia Pacific Uninet: President Prof. Wolf-Dieter Rausch (**c** left) and Prof. Gerhard Litscher (**c** right) with representatives from the Dance Academy, Beijing, China, 20 November 2015.

**Figure 32:**
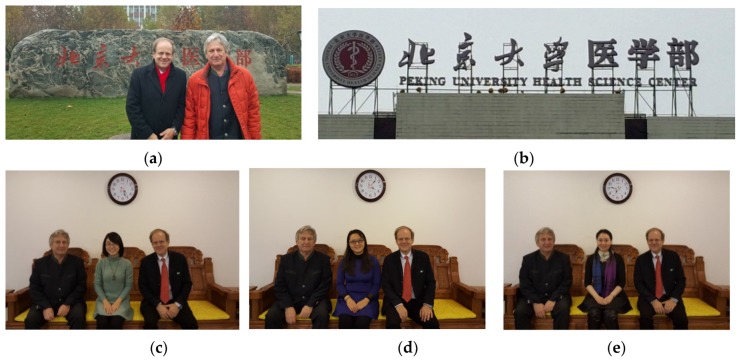
Prof. Wolf-Dieter Rausch (**a** right) and Prof. Gerhard Litscher (**a** left) at Peking University Health Science Center (**b**–**e**), Beijing, China, 20 November 2015.

**Figure 33:**
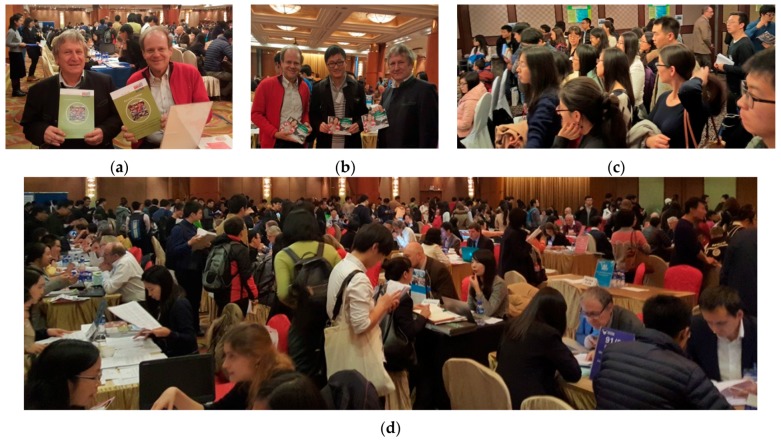
Prof. Wolf-Dieter Rausch (**a** left) and Prof. Gerhard Litscher (**a** right) at the PhD Workshop in Beijing (**a**–**d**), China, 21 and 22 November 2015.

**Figure 34:**
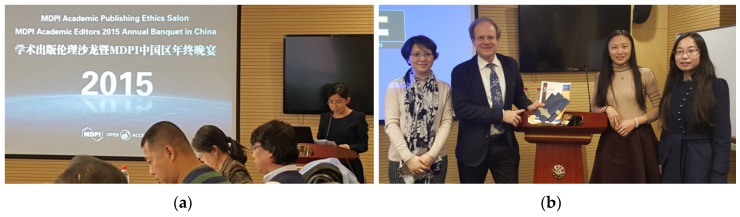
Workshop and annual banquet in Beijing (**a**). Gerhard Litscher (**b** second from left), editor-in-chief of *Medicines*, and Gao Xinyan (**b** left), associate editor of *Medicines* together with representatives from *Medicines*.

**Figure 35:**
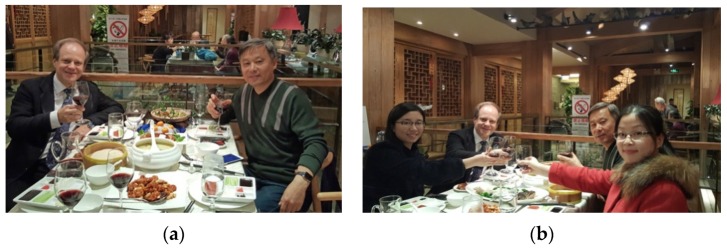
Prof. Shi Xian (**a** right) and Prof. Gerhard Litscher (**a** left), discussion; clinical study: acupotomy (**b**).

**Figure 36:**
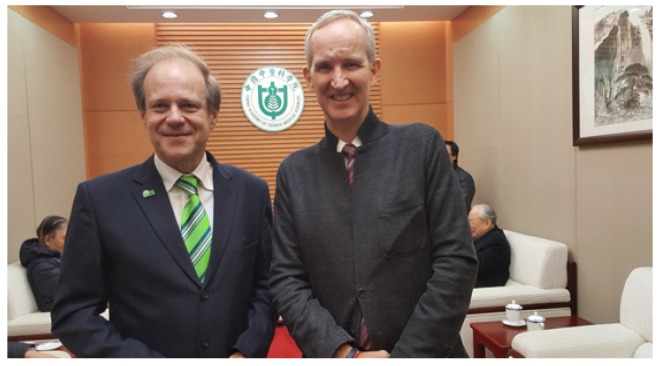
Dr. Bernhard Schwartländer (right), representative of World Health Organization and Prof. Gerhard Litscher (left), Beijing, China, 22 December 2015.

**Figure 37:**
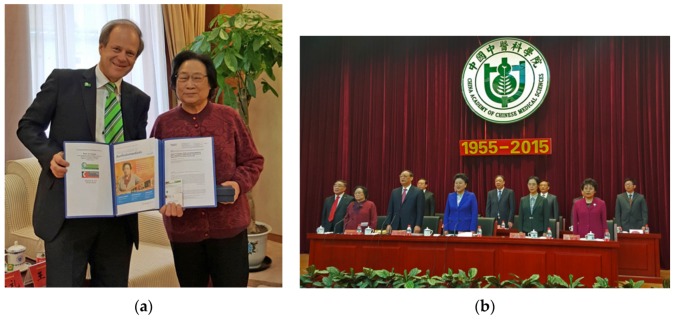
Prof. Tu Youyou (**a** right) and Prof. Gerhard Litscher (**a** left), Beijing, China, 22 December 2015, Celebration with Vice Premier Minister Liu Yandong (**b** middle).

**Figure 38:**
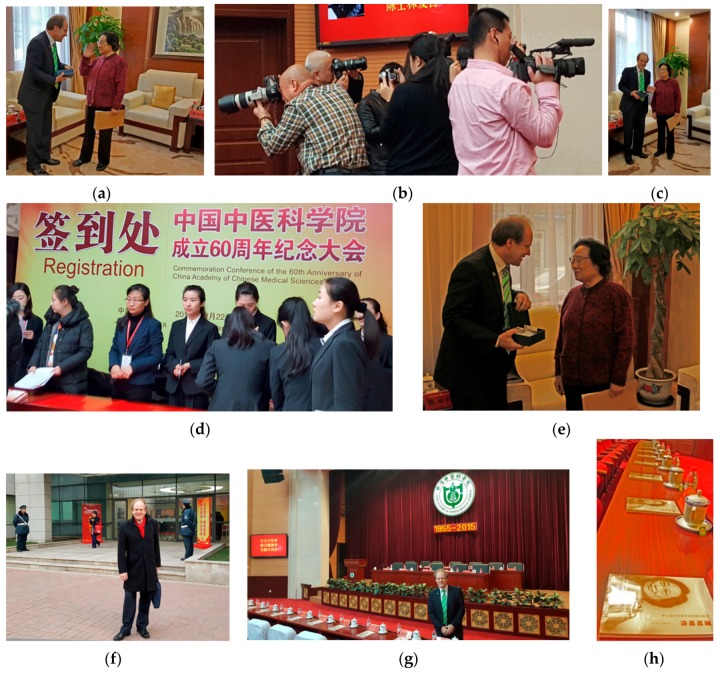

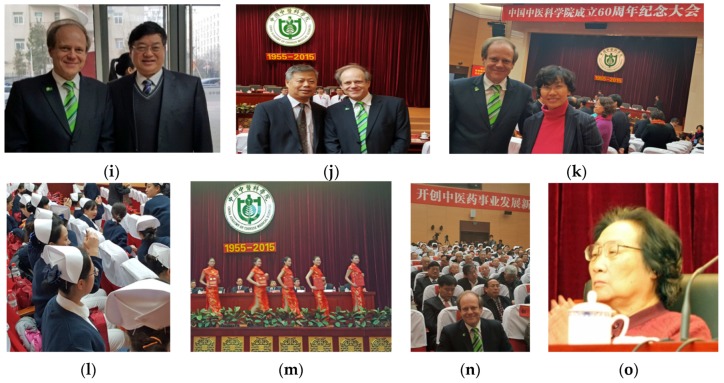
Impressions, 60th Anniversary of China Academy of Chinese Medical Sciences (**a**–**o**). Prof. Tu Youyou (**a**,**c**,**e** right, **o**) and Prof. Gerhard Litscher (**a**,**c**,**e** left), Beijing, China, 22 December 2015.
